# Screening of free carnitine and acyl-carnitine status in patients with Familial Mediterranean Fever

**DOI:** 10.1186/1546-0096-13-S1-P77

**Published:** 2015-09-28

**Authors:** E Kiykim, K Barut, AC Aktuglu-Zeybek, T Zubarioglu, MS Cansever, A Aydin, O Kasapcopur

**Affiliations:** 1 Istanbul University, Cerrahpasa Medical Faculty, Pediatric Metabolic Diseases, Istanbul, Turkey; 2 Istanbul University, Cerrahpasa Medical Faculty, Pediatric Rheumatology, Istanbul, Turkey

## Introduction

Familial Mediterranean fever (FMF) is an autosomal recessive disease characterized by recurring self-limited fever, abdominal pain and chest pain caused by serositis. FMF mainly affects Middle-East populations with a high prevalence in Sephardic Jews, Turkish, Arabs and Armenians. Carnitine is an important molecule in cellular energy metabolism. Secondary carnitine deficiency can be detected in chronic diseases by either renal loss or increased needs.

## Objectives

Our hypothesis was that FMF patients would have lower free carnitine levels than their healthy age and gender matched controls due to increased need of carnitine because of recurrent auto-inflammation. The present study was conducted to determine the patterns of free carnitine and acyl-carnitine esters in FMF patients.

## Methods

This is a cross-sectional study of 205 FMF patients who were attending the outpatient Pediatric Rheumatology clinic of Cerrahpasa Medical Faculty Children's Hospital. The patients were selected by random sampling and FMF diagnosis was confirmed by a pediatric rheumatologist according to Yalcınkaya criteria. 50 healthy subsects were enrolled to the present study. A fasting died blood sample was taken for studying free carnitine and acyl-carnitine esters with tandem mass spectrometry from children in both groups.

## Results

Acyl-carnitine analyses in spot dried blood samples with ESI-MS/MS were performed in all patients and control group. Screening of acyl-carnitine profile revealed free carnitine, C16-OH and C18:2 carnitine levels were higher (p<0,0001, p<0,0001 and p-0,003 respectively), while C4-OH and C4DC carnitine levels were lower (p<0,0001) in FMF patients than the control group.

## Conclusions

In the present study we were not able to define secondary carnitine deficiency in FMF patients, therefore usage of carnitine in all patients with FMF is not recommended.

